# Expansion of brucellosis detection in the country of Georgia by screening household members of cases and neighboring community members

**DOI:** 10.1186/s12889-015-1761-y

**Published:** 2015-05-02

**Authors:** Lia Sanodze, Christian T Bautista, Natalia Garuchava, Svetlana Chubinidze, Ekaterine Tsertsvadze, Mariam Broladze, Nazibrola Chitadze, Ketevan Sidamonidze, Shota Tsanava, Tamar Akhvlediani, Robert G Rivard, Rupal Mody, Matthew J Hepburn, Philip H Elzer, Mikeljon P Nikolich, Nino Trapaidze

**Affiliations:** National Center for Disease Control and Public Health, Tbilisi, Georgia; Walter Reed Army Institute of Research, Silver Spring, MD USA; I. Javakhishvili Tbilisi State University, Tbilisi, Georgia; U.S. Army Medical Research Unit - Georgia, Tbilisi, Georgia; U.S. Army Medical Research Institute of Infectious Diseases, Fort Detrick, MD USA; School of Animal Sciences, Louisiana State University AgCenter, Baton Rouge, LA USA

**Keywords:** Brucellosis, Epidemiology, Zoonotic, Surveillance, Country of Georgia

## Abstract

**Background:**

Brucellosis is considered as endemic zoonotic disease in the country of Georgia. However, the burden of the disease on a household level is not known. Therefore, this study sought to determine the benefits of active surveillance coupled to serological screening for the early detection of brucellosis among close contacts of brucellosis cases.

**Methods:**

We used an active surveillance approach to estimate the rate of seropositivity among household family members and neighboring community members of brucellosis index cases. All participants were screened using the serum tube agglutination test (SAT). Blood cultures were performed, obtained isolates were identified by a bacteriological algorithm, and confirmed as *Brucella spp.* using real-time PCR. Further confirmation of *Brucella* species was done using the AMOS PCR assay.

**Results:**

A total of 141 participants enrolled. Of these, 27 were brucellosis index cases, 86 were household family members, and 28 were neighboring community members. The serological evidence of brucellosis in the household member group was 7% and the rate at the household level was 21%. No screened community members were *Brucella* seropositive. Majority of brucellosis cases were caused by *B. melitensis*; only one index case was linked to *B. abortus*.

**Conclusion:**

We found evidence of brucellosis infection among household family members of brucellosis index cases. *B. melitensis* was the most common species obtained. Findings of this active surveillance study highlight the importance of screening household family members of brucellosis cases and of the use of culture methods to identify *Brucella* species in the country of Georgia.

## Background

Brucellosis is a febrile, debilitating worldwide zoonotic illness caused by Gram-negative coccobacilli of the genus *Brucella* [1]. Human brucellosis is usually linked to ingestion of unpasteurized dairy products of infected ruminant livestock or direct contact with infected animal parts, with inoculation through skin and mucous membranes and more rarely through the inhalation of aerosolized particles [2]. In Georgia, a country in the Caucasus region brucellosis is endemic, but there are still many unanswered epidemiological and clinical questions regarding disease [3,4]. For instance, the burden of the disease among close contacts of brucellosis cases is not known. Epidemiologically, knowledge of contact patterns is critical to design effective control measures for endemic diseases because it allows identification of specific groups in a population for public health planning [5].

Georgia currently uses a passive surveillance approach for brucellosis. This type of surveillance is subject to multiple limitations including underreporting [6]. Several studies conducted in brucellosis-endemic areas have shown the importance and benefits of active surveillance by screening household family members of brucellosis cases [7,8]. In addition, the implementation of an active surveillance program can significantly enhance early disease detection to provide better disease incidence estimates and reduce disease complications. From a population perspective, active surveillance studies have reported high rates of infection among household contacts of brucellosis cases in endemic countries such as Saudi Arabia (19%), Iran (20%), Peru (8%), and recently in Azerbaijan (10%) [9-12].

The aim of this active surveillance study was to determine the burden of brucellosis infection among household family members and neighboring community members of patients with brucellosis in the country of Georgia.

## Methods

### Study population

Between May 2009 and July 2011, individuals 18 years of age or older with confirmed brucellosis at the Medical Parasitology and Tropical Medicine Research Institute (MPTMRI) in Tbilisi were invited to participate as brucellosis index cases for this study. A confirmed brucellosis case was defined as having a compatible clinical symptomatology with an epidemiological link plus a positive laboratory result. A compatible symptomatology was defined as a fever (>38°C) for at least five days and at least two of the following signs or symptoms: sweats, rigors, malaise, fatigue, anorexia, weight loss, arthralgia, myalgias, arthritis, neuritis, neuro-psychiatric symptoms, epididymo-orchitis or changes in liver function tests. An epidemiological link suggestive of brucellosis included assistance with animal birth, involvement in animal husbandry, contact with sick animals, consumption of unpasteurized milk or dairy products, or consumption of undercooked meat. A positive laboratory finding was defined as a titer ≥ 1:200 by the serum tube agglutination test (SAT) [13] and/or isolation of *Brucella* spp. from blood culture [14]. SAT was performed using serial (*x*2) dilutions of serum samples and *Brucella abortus* Antigen (BD), following manufacturer’s instructions. *Brucella* Positive Control (BD) and Febrile Negative Controls (BD) were utilized to assess the test performance. Written informed consent was obtained from brucellosis cases to participate in the study as an index case and to approach their household family members for brucellosis testing. Then they were administered a standardized questionnaire to collect socio-demographic and epidemiological data, as well as exposure history and clinical information associated with brucellosis.

### Measures

For this study, a field team composed of an epidemiologist and a phlebotomist visited the brucellosis index case household to enroll the household family members and neighboring community members. A household family member was defined as an adult or a child (≥5 years old), who consumed at least five meals per week in the same house as the brucellosis index case and/or was living in the same household for at least two months prior to enrollment. For each brucellosis index case, a neighboring community household was systematically selected (three houses away, either to the right or left of the index house). A neighboring community member was defined as an individual five years old or older. For both groups, household and community, all individuals meeting selection criteria were invited to voluntarily participate. Volunteers were enrolled after obtaining written consent. A blood sample was drawn at enrollment, and then again after 2-4 weeks at a follow-up visit. The same questionnaire, used among brucellosis index cases was applied to enrolled household and community members to collect epidemiological data associated with brucellosis. A microbiological testing algorithm and real-time polymerase chain reaction (PCR)-based methods (Target 1, Idaho Technology Inc.) were used to identify *Brucella* blood culture isolates, while the conventional AMOS PCR assay was used to confirm the *Brucella* species [14,15].

Several articles have reported the successful use of PCR for the detection of *Brucella*-specific DNA in blood and serum samples using various platforms [16-20]. This approach provides a rapid tool to confirm the presence of *Brucella*. In attempt to identify a reliable and reproducible approach for detecting *Brucella*-specific DNA directly from clinical samples several real-time PCR assays (targeting *Brucella* T1 [Idaho Technology Inc.], B4/B5, IS*711*) were tested using various DNA isolation methods and amplification conditions. Although preliminary experiments using spiked samples provided promising results, no reproducible and definite amplification was obtained when using blood or serum samples from acute phase of culture-positive subjects (*publication in preparation*). Thus, only culture and SAT results were reported.

### Ethical approvals

This study protocol was reviewed and approved by institutional review boards at the U.S. Army Medical Research Institute of Infectious Disease, Ft. Detrick, MD (HP-08-25), Walter Reed Army Institute of Research, Silver Spring, MD (WRAIR #1866), and at the National Center for Disease Control and Public Health, Tbilisi, Georgia.

## Results

**Figure 1:**
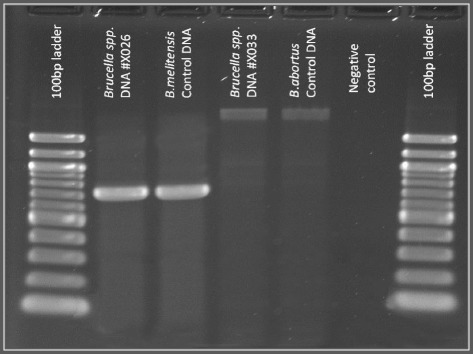
Amplification of DNAs of *Brucella spp* isolates by AMOS PCR. From left to right: 100 bp ladder (*Sigma Aldrich*); Isolate X026, *B. melitensis*, *B. melitensis* Positive control, Isolate X033, *B. abortus*, *B. abortus* Positive control, Negative control (no DNA), 100 bp ladder.

During the study period, a total of 141 participants were enrolled. Of these, 27 were brucellosis index cases, 86 were household family members of index cases, and 28 were neighboring community members. Ninety-three percent (n = 25) of brucellosis index cases were positive for *Brucella*-specific antibody by SAT (6 cases, 1:200; 3 cases, 1:320; 9 cases, 1:400; 5 cases, 1:640; 1 case, 1:800, and 1 case, 1:1280). Of these, 11 isolates identified by the bacteriological algorithm were confirmed as *Brucella* spp. by real-time PCR. Furthermore, the AMOS PCR assay confirmed that 10 isolates belonged to *B. melitensis* and one sample to *B. abortus* (Figure 1). Additionally, two brucellosis index cases were serologically negative by SAT at enrollment but *B. melitensis* was isolated, and thus these were subsequently included in the group of index cases.

The median age of brucellosis index cases was 35 years (range: 19-63), 93% were males, 93% lived in rural areas, 67% had high school education, 41% were farmers, 41% were from Kakheti, and 35% from Kvemo-Kartil regions (eastern Georgia). The most common signs and symptoms after fever for brucellosis index cases were sweats (100%), fatigue (96%), joint pain (93%), malaise (93%), rigors (88%), and to lesser extent back pain (35%), and muscle pain (30%). All brucellosis index cases reported having consumed either of fresh cheese, yogurt, milk, sour cream or sheep cheese in the past four months. They also reported having consumed unpasteurized dairy products (88%) or not boiled milk (15%), using home-made dairy products (67%), and slaughtering at home (59%). Most brucellosis index cases had livestock in their households (81%, sheep, cattle or goats), 63% had direct contact with sick animals, and 43% with aborted livestock in the past four months. Regarding self-protection against brucellosis, 26% had knowledge about disease, 36% washed their hands after handling animals, and none wore gloves when handling animals (Table 1).

**Table 1:** **Characteristics of brucellosis index cases, household family members, and neighboring community members**

**Feature**	**Index cases (N = 27)**	**Household members (N = 86)**	**Community members (N = 28)**
	**n (%)**	**n (%)**	**n (%)**
Age (years), mean (SD)	36.9 (12.1)	33.8 (20.3)	46.1 (16.6)
Gender, men	25 (93)	37 (43)	11 (39)
Regions, Kakheti and Kvemo-Kartli (eastern)	22 (81)	69 (80)	26 (93)
Consumed milk or dairy products^a^	27 (100)	84 (98)	28 (100)
Consumed unpasteurized dairy products	23 (88)	59 (69)	18 (69)
Not boiling milk before consumption	5 (15)	32 (27)	8 (29)
Slaughter at home	16 (59)	35 (41)	12 (43)
Occupational exposure^b^	23 (85)	23 (27)	8 (29)
Direct contact with sick animals	12 (63)	17 (34)	2 (13)
Brucellosis knowledge	7 (26)	28 (33)	10 (36)
Wash hands after handling animals	9 (36)	46 (68)	17 (89)
Wear gloves with animals birthing	0 (0)	3 (3)	1 (3)
Self-protect from brucellosis	2 (7)	5 (6)	1 (4)
Vaccinate animals against brucellosis	1 (4)	7 (10)	5 (24)

NOTE: SD = standard deviation.

^a^Milk, yogurt, sour cream, fresh cheese, or sheep cheese.

^b^Cut animals’ carcasses, peeled animals’ skin, slaughter cattle, assisted cattle in delivery or abortion.

Denominators may vary due to missing data.

In the household family member group, the median age was 27 years. Forty three percent of this group was male, 41% had high school education, and 35% were housewives. Over 73% made their dairy products at home, 69% consumed unpasteurized dairy products, and 37% did not boil milk before consumption. They also reported having livestock in the household (87%) and direct contact with sick (34%) or aborted livestock (11%) in the past four months. Moreover, one-third of them had knowledge about the disease, 68% washed hands after handling animals, and only 7% reported wearing gloves when handling animals.

At enrollment, four household members had serological titers ≥1:320 for *Brucella*. Additionally, two household family members without any clinical manifestations were serologically negative by SAT but positive by blood culture (Table 2). The brucellosis rate among household family members was 7.0% (6/86; 95% confidence interval = 2.9-13.9%), and the brucellosis rate at the household level was 21% (6 out of 27 households had at least one member with brucellosis). Four of the six household family members found to be positive by SAT or by blood culture returned for a follow-up visit, and were symptomatic. The other two cases refused to continue participation thus no follow-up information is available. No epidemiologic risk factor was associated with brucellosis infection in the household family member group.

**Table 2:** **Demographic characteristics, risk factors, and serum agglutination test and culture isolated of six brucellosis cases found among household family members of brucellosis index cases**

**ID**	**Age (yrs)**	**Sex**	**Region**	**Occupation**	**Consumed unpasteurized dairy products**	**Slaughter at home**	**Boil milk**	**Wear gloves when handling animals**	**SAT**	**Culture isolated**
X04-C1	33	F	Kvemo Kartli	housewife	Yes	Yes	Yes	No	1:640	Neg
X13-C2	16	M	Mtskheta-Mtianeti	student	Yes	Yes	No	Yes	Neg	*B. melitensis*
X14-C9	8	M	Kakheti	other	Yes	Yes	Yes	No data	Neg	*B. melitensis*
X19-C1	48	M	Kvemo Kartli	other	No	No	No	No data	1:640	Neg
X28-C3	62	F	Kvemo Kartli	unemployed	Yes	No	No data	No	1:320	Neg
X30-C2	27	M	Kvemo Kartli	unemployed	Yes	No	Yes	No data	1:320	Neg

Note: F = female; M = male; SAT = serum agglutination test.

In the neighboring community group, the median age was 46 years, 39% were males, 43% had high school education, 54% were housewives; and all lived in rural areas. Over 29% did not boil milk before consumption, 69% consumed unpasteurized dairy products, 61% made dairy products at their homes, and 43% did home slaughtering. Sixty-one percent had livestock in the household, 13% had direct contact with sick livestock, and none reported having contact with aborted livestock in the past four months. Most neighboring community members washed their hands after handling animals (89%), but few (8%) reported wearing gloves when handling animals. Additionally, 36% reported having knowledge of brucellosis. No community member was found to be *Brucella* seropositive by SAT or by blood culture.

## Discussion

In this study, an active surveillance approach was used to obtain evidence of *Brucella* infection among household family members of brucellosis index cases. The brucellosis rate among household family members was similar to that reported in Peru, Turkey, and recently in the neighboring country of Azerbaijan [7,11,12]. Our findings were consistent with other previous studies addressing the benefits and importance of screening household members of brucellosis index cases in endemic areas [7-12]. Such screening efforts may help reduce the burden of brucellosis and its medical complications, as well as improve treatment outcomes in the country of Georgia.

An interesting finding from this study was the detection of two culture-confirmed brucellosis cases among household family members without any clinical manifestation and serologically negative by SAT. This suggests that SAT, the most widely used method for the laboratory diagnosis of human brucellosis, is not sensitive enough to detect antibodies to *Brucella* species in subjects with early bacteremia. It is possible that during the active stage of the disease, antinuclear antibodies be a reason for false negative results by SAT [21]. It is also noteworthy that SAT is currently the main laboratory tool supporting confirmation of clinical diagnosis of human brucellosis in Georgia.

We believe that the introduction of additional screening and confirmatory laboratory testing - for instance, culture combined with reliable and reproducible PCR methods -can strengthen the *Brucella* detection capacity in the country of Georgia. In addition to detecting the *Brucella* organisms by culture and PCR, results from these methods can be also used to evaluate drug regimens for treatment of brucellosis [22].

The failure of PCR to detect *Brucella*-specific DNA directly in clinical samples (blood and serum) is supported with recent suggestions in the literature and held within the brucellosis research community that the sensitivity and the specificity of most PCR-based methods are not well established. The real value for use of PCR diagnostics with clinical samples, and hence for diagnosis, has not yet been properly validated [23]. Considerable laboratory work is still required to verify, validate, and establish standard positive and negative controls, internal and inhibition controls, quality assurance and contamination control before PCR could be considered for a routine test for brucellosis diagnosis.

In our study, the overall brucellosis male-to-female ratio was about 8:1. This high gender disparity may be due to occupational exposure differences, since males usually work on the farms and in the care and management of farm animals [24]. Thus, brucellosis is more likely to occur in males than in females in Georgia. This fact was observed in this study, where 93% of brucellosis index cases were males, and 4 out of 6 cases identified in the household family member group were males (Table 2). A similar finding was reported in a previous brucellosis study conducted in Azerbaijan [12]. We have also found that the majority of the brucellosis index cases seeking medical care at MPTMRI were residents of rural areas, mainly from the Georgian regions of Kvemo-Kartli (southeastern) and Kakheti (eastern). Sheep husbandry and shepherding are common activities among rural farmers from eastern Georgia [3,4].

*B. melitensis* is responsible for the most severe brucellosis infection in humans and is more common in Latin America, the Mediterranean area, Central Asia, and in the Caucasus region [25]. The main reservoirs for *B. melitensis* are sheep and goats. Human infection with this species is commonly acquired through ingestion of contaminated dairy products or by direct contact with infected animals or animal discharges [26]. In this study, almost all of the brucellosis cases were caused by *B. melitensis*. Our epidemiological data suggest that occupational exposure to animals is probably the most common cause of human transmission for this *Brucella* species among male brucellosis index cases and male household family members. In addition, *B. melitensis* appears to be the predominant species associated with human brucellosis in eastern Georgia.

Interestingly, the data on exposure to modes of *Brucella* transmission was similar among neighboring community members and household family members (Table 1), while no brucellosis cases were found among community members in the study. We hypothesize that the absence of brucellosis in the community group might be associated with the practice of some preventive measures to control human and animal brucellosis [27]. Neighboring community members compared to their counterparts reported higher percentages for hand washing after handling animals, animal vaccination against brucellosis, and boiling milk before consumption. To our knowledge, there is insufficient evidence on the effectiveness of preventive measures against brucellosis in Georgia. Therefore, further studies are needed to determine whether common preventive measures along with other barriers methods not addressed in this study can help minimize or probably eliminate the risk of human brucellosis transmission.

This study had some limitations. First, the small number of female brucellosis cases limited the statistical power to detect risk factors in this group of participants. It is believed that in the Caucasus region, consumption of unpasteurized dairy products constitutes an important mechanism of brucellosis transmission, mainly among women and children; however, the burden of this probable source of *Brucella* infection and its public health implication are still not determined. Second, no risk factor was associated with exposure to *Brucella* among household family members of brucellosis index cases. This lack of association may be attributed to the small number of cases found in this group. Third, the number of community members was relatively small due to non-participation, and thus, the burden of disease in this group may be biased. Finally, most brucellosis cases at MPTMRI were from eastern Georgia, thus our results may not represent the true situation of brucellosis in other regions of the country such as west Georgia. Despite these limitations, it is noteworthy that this study was the first attempt to use an active surveillance approach to evaluate the importance of screening household family members and neighboring community members for brucellosis in the country.

## Conclusions

In summary, using an active surveillance approach for brucellosis we found evidence of *Brucella* infection among household family members of brucellosis index cases, and the high ratio of male-to-female cases suggests the disease is largely occupational. Moreover, almost all brucellosis cases from eastern Georgia were caused by *B. melitensis*. An active surveillance approach enhances disease detection and provides additional epidemiological data that can be used to behavior change interventions in order to reduce the incidence of human brucellosis in Georgia.
